# Immune Profile Analysis in Peripheral Blood and Tumor in Patients with Malignant Melanoma

**DOI:** 10.3390/ijms22041957

**Published:** 2021-02-16

**Authors:** Ryuichi Saito, Yu Sawada, Motonobu Nakamura

**Affiliations:** Department of Dermatology, University of Occupational and Environmental Health 1-1, Iseigaoka, Yahatanishi-Ku, Kitakyushu 807-8555, Japan; rs.med3110@gmail.com (R.S.); motonaka@med.uoeh-u.ac.jp (M.N.)

**Keywords:** melanoma, immune checkpoint, anti-PD1 antibody, nivolumab, immune cells

## Abstract

Melanoma is a severe and life-threatening malignancy derived from melanocytes. The traditional treatment for melanoma could not sustain satisfactory outcomes long term; however, the recent immune checkpoint treatment has made a breakthrough in these problems. Nivolumab is a representative immune checkpoint treatment, and this PD-1-targeted therapy has evolutionally developed and improved the clinical outcome in a recent decade. On the other hand, the clinical application of immune checkpoint treatment presents clinicians with novel questions, especially how to obtain additional efficacy and overcome the disadvantage by using this treatment. To answer these problems, we first investigated the distribution of PD-L1 in various organs to clarify the organs most affected by anti-PD-1 antibody treatment. Among various organs, lung, placenta, spleen, heart, and thyroid highly expressed PD-L1, while skin, thalamus, hippocampus, ovary, stomach, testis, and prostate showed lower expressions of PD-L1. Furthermore, the immune profiles were also examined in tumors and peripheral blood in patients with melanoma. PD-1 was highly expressed in CD8 and CD4 cells, and B cells also highly expressed PD-1 compared with NK cells. However, there was no significant difference in Th1/Th2/Th17 cytokines and inhibitory cytokine IL-10. Although nevus showed a low expression of PD-L1 compared with healthy skin, PD-L1 expression was increased in growth-phase melanoma. Finally, we analyzed the peripheral blood profiles in patients treated with nivolumab. PD-1-bearing dendritic cells (DCs) were increased during nivolumab treatment and Lin-CD11c+HLA-DR+ cells were highly increased during nivolumab treatment. These findings indicate a clue to answering the problems during nivolumab treatment and suggest to us the importance of multiple aspect observation during immune checkpoint treatment.

## 1. Introduction

Melanoma is a malignancy derived from melanocytes with an unfavorable clinical behavior because of the characteristics of severe and life-threatening malignancy [1]. However, the recent development of immune checkpoint therapy for melanoma has dramatically improved clinical outcomes in advanced melanoma [1,2,3]. Therefore, immune checkpoint therapy is expected to become an important treatment in the advanced stage of various malignant tumors as a current trend. On the other hand, the clinical application of immune checkpoint treatment presents clinicians with novel questions, especially how to obtain additional efficacy and overcome the disadvantage by using this treatment.

Nivolumab is a representative immune checkpoint treatment for advanced melanoma and shows favorable outcomes even through its advanced clinical stages [3]. On the other hand, immune checkpoint therapy sometimes causes immune-related adverse events (irAEs) by the cancellation action of immune escape phenomenon via PD-1/PD-L1-mediated mechanisms [4,5]. Actually, PD-1 deficiency exacerbates autoimmune reactions. PD-1-deficient mice developed a lupus-like autoimmune disease [6] and an autoimmune dilated cardiomyopathy [7]. From these findings, it is apparent that PD-1-targeted treatment exacerbates these autoimmune diseases against host human organs. Thus, the question of how to suppress these side effects is one of the highlighted issues for clinicians. It is known which liable organs cause these adverse events [5]. As one of the mechanisms, organs bear PD-L1 on their cell surfaces and directly act on immune cells, especially T cells, to escape from the immune response causing autoimmune reactions [8]. On the basis of this theory, the degree of PD-L1 on organ tissues contributes to the regulation of the autoimmune reaction against immune cells during immune checkpoint therapy. However, a detailed analysis of the PD-L1 expression on each organ in the human body has yet to be performed.

Melanoma is an immunogenic malignancy and mediates immunosuppressive action. For instance, vaccination for melanoma fails to affect the tumor growth despite the presence of reactive T cells against melanoma in peripheral blood [9,10,11,12]. Furthermore, possibilities of immunosuppressive mechanisms by inhibitory cytokines and regulatory T cells-mediated immunosuppressive mechanisms in melanoma have been postulated [13]. Therefore, these melanoma-mediated immunosuppressive actions suppose that melanoma carrier conditions might establish an imbalance in immune profiles in the tumor and peripheral blood, reflected as local and systemic immune profiles, respectively. However, it remains largely unknown to what degree melanoma-mediated immunosuppression influences human host immune profiles. Furthermore, the characteristics of responders and non-responders to nivolumab are important issues during nivolumab treatment. Although recent advancements in our understanding of the nivolumab treatment response in melanoma patients have been made, the verification of these data by other studies is necessary to further develop current research fields.

In this study, we focused on these problems during anti-PD-1 antibody treatment. First, we analyzed PD-L1 expression in various organs. Further, we also examined PD-1/PD-L1 expression and immune profiles in peripheral blood and melanoma tissues. Finally, we checked the characteristics of responders and non-responders during nivolumab administration to confirm previously published research. These findings are helpful for the management of melanoma during nivolumab administration.

## 2. Results

### 2.1. The Distribution of PD-1 and PD-L1 Expression in Human Organs

**Figure 1:**
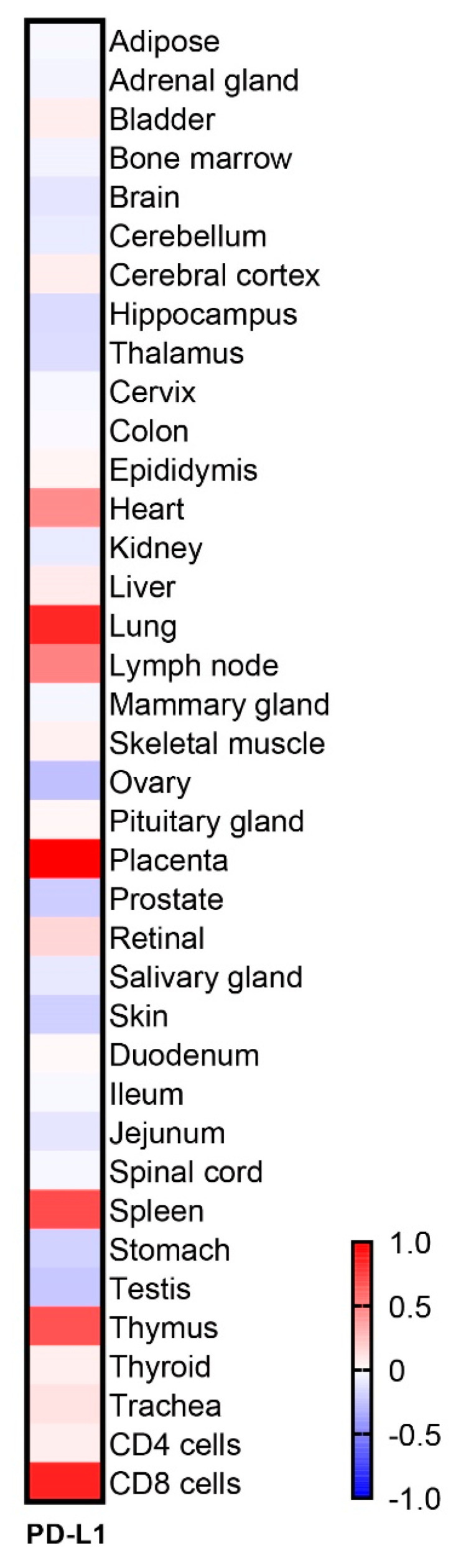
The distribution of PD-L1 in various healthy human organs. Heatmap to visualize the expression of PD-L1 by an RNA-seq data set analysis.

As one of the reasons for self-defense against an autoimmune reaction, it was assumed that the distribution of PD-L1 might contribute to the suppression of organ-specific autoimmune diseases. To clarify this, we analyzed PD-L1 expression on various organs by using public data sets of RNA-seq. Among various organs, lung, placenta, spleen, heart, and thyroid highly expressed PD-L1 Figure 1, while skin, thalamus, hippocampus, ovary, stomach, testis, and prostate showed lower expressions of PD-L1. Therefore, the degree of PD-L1 expression was different depending on each organ.

### 2.2. Peripheral Blood Expression of PD-1 and Immune Profiles in Melanoma

**Figure 2:**
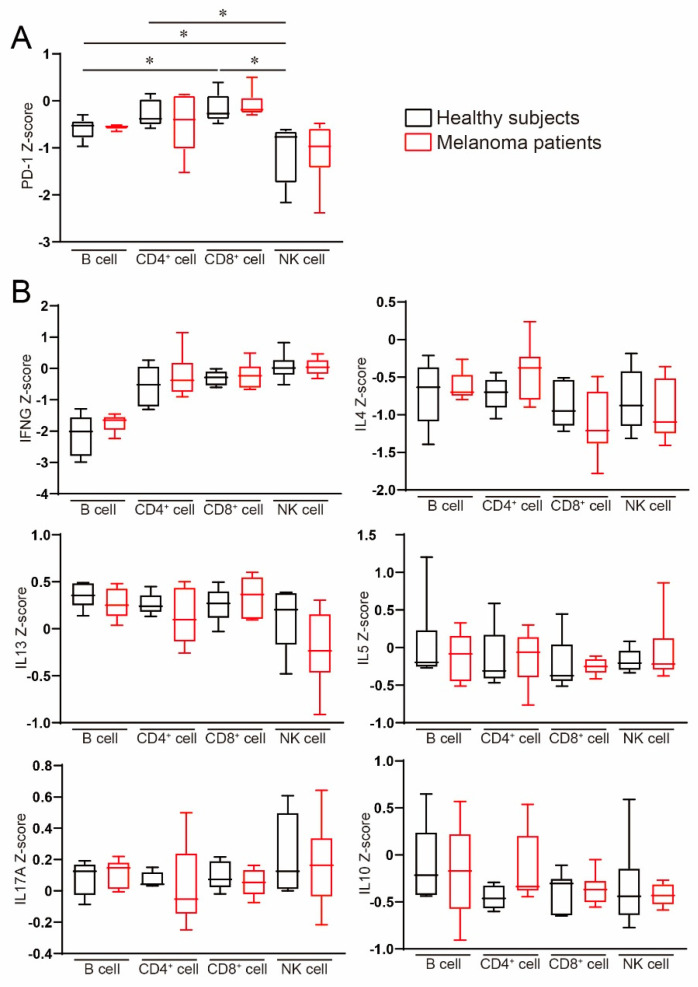
Gene expression in peripheral blood immune cell subsets. RNA-seq data set analysis in peripheral blood immune cells in healthy subjects and melanoma patients. (**A**) PD-1 and (**B**) inflammatory cytokines were evaluated. Results are expressed as the mean ± SE. All *p*-values were obtained by Student’s *t* test: *, *p* < 0.05.

Next, we investigated the difference in PD-1/PD-L1 expression in peripheral blood immune cell subsets by using a public data set. PD-1 was highly expressed in CD8 and CD4 cells, and B cells also highly expressed PD-1 compared with NK cells Figure 2. We speculated that a melanoma-mediated immunosuppressive effect might systemically influence immune cells, especially peripheral blood. Therefore, we also analyzed the differences in immune profiles between healthy subjects and melanoma patients; however, there was no significant difference in neither Th1/Th2/Th17 cytokines nor inhibitory cytokine IL-10 Figure 2. Therefore, these findings suggest that melanoma-bearing conditions might not affect systemic immunosuppression mediated by melanoma.

### 2.3. Tumor Site Expression of PD-1/PD-L1 and Immune Profiles

**Figure 3:**
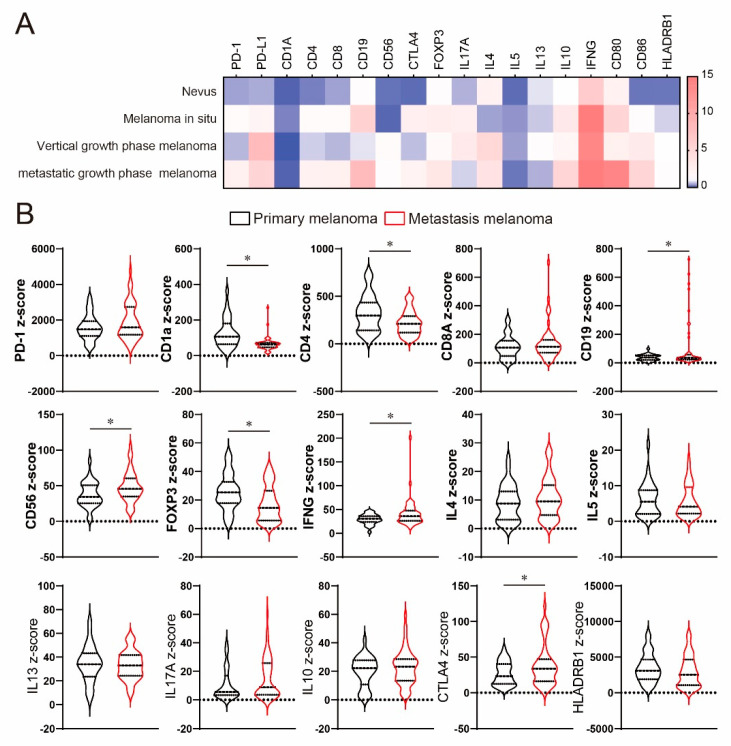
Gene expression in the tumor in patients with melanoma. (**A**) Heatmap representing relative gene expression compared with healthy skin. (**B**) Violin plots showing the differences in gene expression in primary melanoma and metastatic melanoma. All *p*-values were obtained by Student’s *t* test: *, *p* < 0.05.

Because our analysis could not find the systemic influence of immune profiles due to melanoma-bearing conditions, we next analyzed local site immune profiles by using the previously published data set. To clarify the difference, we first visualized the difference between healthy skin, nevus, melanoma in situ, and growth-phase melanoma. While nevus showed a low expression of PD-L1 compared with healthy skin, PD-L1 expression was increased in growth-phase melanoma Figure 3A. Dendritic cell (DC) marker CD1a was decreased in patients with melanoma and nevus. On the other hand, CD80 expression was also increased in advanced growth-phase melanoma.

To validate these findings, we also analyzed immune profiles in patients with primary melanoma and metastatic melanoma by using another public data set. There was no significant difference in PD-1 expression between primary and metastatic melanoma Figure 3B. CD4 and the DC surface marker CD1a were significantly decreased in metastatic melanoma, while CD19 (B cell marker) and CD56 (NK cell marker) were increased in metastatic melanoma. In inflammatory cytokines, IFN-γ was increased in metastatic melanoma, while there was no significant difference in IL-4, IL-5, IL-13, and IL-17A. Regulatory T cells are known to be involved in anti-tumor immunity in melanoma patients, and the importance of therapeutic application of CTLA4 is proven in melanoma [14]. Foxp3 expression was decreased in metastatic melanoma, while CTLA4 expression was significantly increased. However, IL-10 expression displayed no significant difference between primary melanoma and metastatic melanoma.

### 2.4. A High Frequency of Lin^−^CD11c^+^HLA-DR^+^-Activated DCs in Responders to Nivolumab

**Figure 4:**
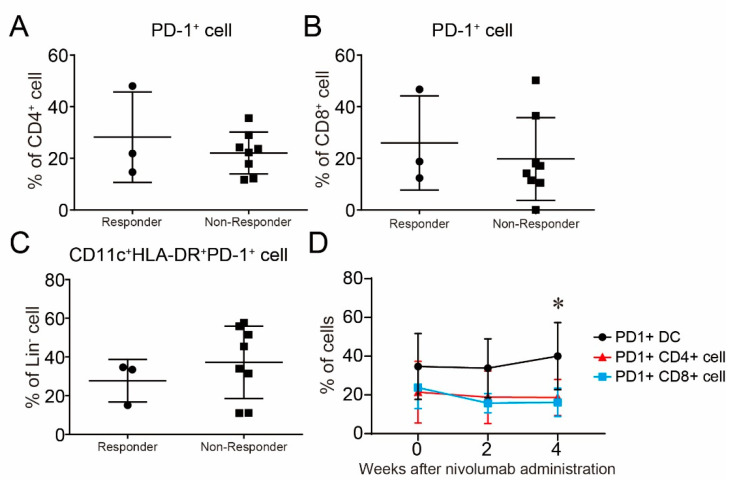
PD-1 expression on immune cells in peripheral blood in patients with melanoma. (**A–C**) PD-1 expression in (**A**) CD4^+^ cells, (**B**) CD8^+^ cells, and (**C**) dendritic cells (DCs) was evaluated in patients with melanoma by FACS analysis to compare the difference between responders and non-responders to nivolumab. (**D**) The time course of PD-1 expression on CD4^+^ cells, CD8^+^ cells, and DCs in all melanoma patients during nivolumab treatment. Results are expressed as the mean ± SE. All *p*-values were obtained by Student’s *t* test: *, *p* < 0.05.

One of the most interesting issues is to clarify the characteristics of the difference between responders and non-responders to nivolumab. To clarify this issue, we first examined the difference in PD-1 expression on peripheral blood mononuclear cells (PBMCs). However, there was no significant difference in PD-1 expression on CD4^+^ cells, CD8^+^ cells, and Lin^−^CD11c^+^HLA-DR^+^ cells between responders and non-responders Figure 4A–C. These results indicate that PD-1 expression on immune cells might not contribute to determining the response to nivolumab and is not useful as a biomarker to predict the responders. However, the frequency of PD-1-bearing DCs was gradually increased compared with T cells after the continuation of nivolumab administration Figure 4D. PD-1^+^ DCs had a higher frequency than PD-1^+^CD4 and PD-1^+^CD8 at baseline; however, there was no significant difference.

**Figure 5:**
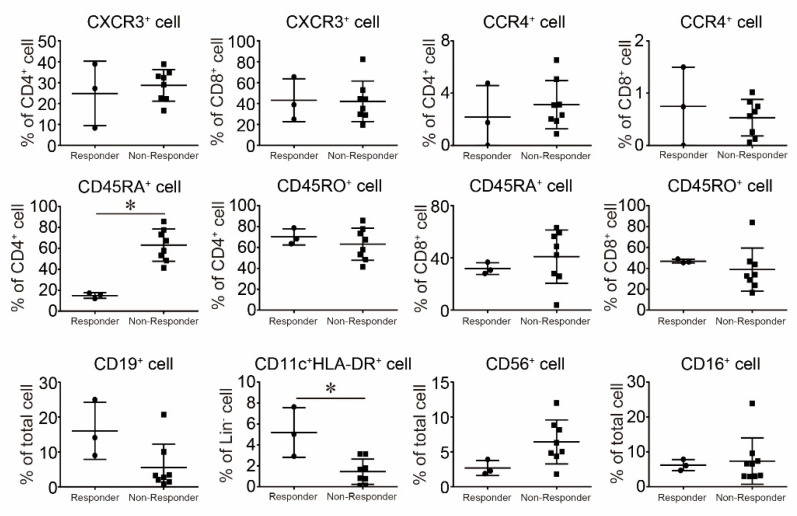
The Comparison of the Frequencies of Immune Cells between Responders and Non-Responders to Nivolumab. The frequencies of each immune cell were evaluated by FACS and we compared the differences in frequency between responders and non-responders to nivolumab. Results are expressed as the mean ± SE. All *p*-values were obtained by Student’s *t* test: *, *p* < 0.05.

Antigen presentation cells are important immune cells for robust anti-tumor immunity. Therefore, we examined the frequency of activated DCs in responders and non-responders. A high frequency of activated DCs, representing Lin^−^CD11c^+^HLA-DR^+^ cells, was observed in favorable responders to nivolumab treatment Figure 5. We also examined the immune profiles in PBMCs between responders and non-responders before the treatment of nivolumab. However, a significant difference could not be observed in Th1/Tc1, Th2/Tc2, and naïve/memory cells between responders and non-responders, excluding CD4^+^CD45RA^+^ cells Figure 5. The frequency of NK cells exhibited a non-significant but marginal difference between responders and non-responders (*p* = 0.0832). Therefore, a high frequency of activated DCs might predict the response to nivolumab. Because the number of patients in Figure 5 is limited, these findings might not provide a solid conclusion. However, these findings support previously published results in part.

Taken together, our results show the characteristics of PD-L1 distribution in organ and immune profiles in melanoma patients. Because some of these data have previously been shown in previous studies, our results can emphasize the importance of these characteristics and suggest that these are concrete findings.

## 3. Discussion

In this study, we clarified the distribution of PD-1 and PD-L1 in various healthy organs. Highly expressed PD-L1 on organs seems to be a critical life component to keep their life in the human body. In fact, these frequencies are relatively low; however, their irAEs cause critical organ disfunction and life-threatening adverse events, such as myositis, interstitial pneumonia, and colitis, during anti-PD-1 antibody treatment. However, it seems that irAEs might not be simply related to the expression of PD-L1 on organs. Skin is an organ with low PD-L1 expression, and cutaneous adverse events are frequently observed during anti-PD-1 antibody treatment. The information on PD-L1 distribution might be helpful to get a better understanding of the mechanisms of irAEs. We also noticed that there were some organs that could not be explained only by the expression of PD-L1. For instance, endocrine glands, such as thyroid and pituitary glands, are organs that frequently cause irAEs during anti-PD1 antibody treatment [4]. These endocrine glands are small in size but actively drive the management of human body homeostasis through small amounts of hormones [15,16,17]. These precise mechanisms might be fragile against an autoimmune reaction. Consistently, anti-PD-1 antibody-related endocrine disorders are often irreversible [18,19]. These findings also suggest to us that organ side vulnerability to autoimmune reactions might be also involved in the pathogenesis of irAEs.

Immune checkpoint therapy for pregnancy is well discussed as one of the important problems and requires difficult decisions in both the malignancy carrier’s (the mother’s) health and the fetus’ development. Several cases reported the efficacy of anti-PD-1 antibody treatment during pregnancy without influencing mother and fetus growth [20,21]. However, nivolumab is a human IgG4 monoclonal antibody [22] that easily passes through the placenta and can reach the fetus. Consistently, PD-1/PD-L1 blockages increase the risk of spontaneous abortions in animal studies [23,24]. Our study showed that the placenta is one of the organs with the highest PD-L1 expression and it is well-established that this organ escapes from autoimmune reactions. This means that PD-1/PD-L1 blockages are critical for the placenta, possibly leading to currently unrecognized influences on fetus development, the same as in animal studies. Furthermore, immune checkpoint treatment for pediatrics increases early phase occurrence of breaking the tolerance against autoimmune reactions [25]. Therefore, long-term evaluation is necessary to clarify the actual impact of fetus development and the future occurrence of autoimmune diseases. Therefore, careful observation is necessary for these patients because organs with high PD-L1 expression also cause an autoimmune reaction during nivolumab treatment; thus, this is not an easily recommended treatment for pregnancy.

In our study, HLA-DR expressed activated DCs that were significantly increased in responders to nivolumab. We speculate that there are two mechanisms of DC contribution during nivolumab treatment. First, because PD-1 is highly expressed on DCs, a blockade of PD-1 signaling by DCs contributes to the development of a T-cell-mediated immune response. Indeed, PD-L1 deficiency on the antigen presentation cells enhanced interferon-γ (IFN-γ) production by T cells [26], and it might easily obtain a favorable response for nivolumab in melanoma patients with highly activated DCs. A recent study reported a correlation of the frequency of CD14^+^CD16^−^HLA-DR^high^ monocytes with a favorable clinical outcome by anti-PD-1 immunotherapy [27]. Therefore, activation of antigen presentation cells might become a therapeutic predictor for melanoma during immune checkpoint therapy.

We found that B cells highly expressed PD-1; however, little is known about the contribution of B cells in melanoma patients. In mouse experiments, depletion of B cells did not affect anti-tumor immunity [28]. Another study showed that the anti-tumor efficacy of melanoma vaccines is enhanced in the absence of B cells [29], and Breg cells contribute to the development of the tumor [30]. On the contrary, during anti-CTLA4 antibody treatment, a significant increase in IL-10 producing B cells was found in the non-responder group [31]. From these current studies, it seems that B cells do not contribute to the suppression of tumor development during anti-PD-1 antibody treatment. NK cells also highly express PD-1 and are well known to contribute to the anti-tumor immunity against melanoma [32,33]. In our study, the difference in NK cell frequency between responders and non-responders was marginal; however, further analysis might clarify the contribution of NK cells during anti-PD-1 antibody treatment.

We also analyzed the difference in inflammatory cytokine expression in melanoma patients. While there were no significant differences in the peripheral blood expression of Th1/Th2/Th17 cytokines, IFN-g slightly increased in metastatic melanoma lesions compared with primary melanoma lesions. As for the immunosuppressive effects of melanoma, CTLA4 was significantly increased in the tumor in patients with metastatic melanoma, consistent with the current efficacy of clinical application of the anti-CTLA4 antibody for advanced melanoma [14]. Because there was no difference in PD-1 expression between responders and non-responders, PD-1 expression might not be suitable to predict the responders to anti-PD-1 antibody treatment. A previous study identified PD-L1 expression on tumors as one of the independent prognostic factors in malignant melanoma; therefore, tumor site analysis might also provide more useful information to predict their prognosis during nivolumab treatment.

The regulatory mechanisms of PD-1 function at the activation stage remain largely unknown. However, a recent study identified that a costimulatory molecule, CD80, interacts with PD-L1 in cis on antigen-presenting cells to regulate PD-L1/PD-1 binding [34]. In our study, CD80 expression was increased as the melanoma growth phase advanced. This means that CD80 is functionally activated and responds to local primary tumor growth. Therefore, further investigation is necessary to develop an understanding of how DCs are activated in melanoma-bearing conditions.

As tumor side variables, responders to anti-PD-1 antibody treatment are associated with tumors bearing PD-L1 [35,36,37] and MHC-class II [38]. On the contrary, as a characteristic of host immune cell profiles in responders, a high frequency of CD4 and CD8+ T cells [35,38], NK cells [39], Th9 [40], central memory cells [41], and effector memory T cells [42] has been observed. IFN-γ is also observed in responders [43,44]. PD-1 expression on immune cells and PD-L1 expression on macrophages are increased in responders [35]. Although our study made a solid conclusion, our results support previous study findings in some parts.

We reported the profiles of PD-1/PD-L1 and immune cell profiles in various organs, tumors, and peripheral blood that are involved in melanoma treatment. Immune checkpoint treatment will become one of the main streams for advanced malignancies. Therefore, the question of how to obtain additional outcomes by immune checkpoint inhibitor treatment, and how it impairs the irAE reaction during the treatment, are hot topics in this current field. PD-1-mediated immune reaction establishes a diverse and complicated network in human immunity. Therefore, multiple-aspect-based observation will be necessary to overcome these problems.

## 4. Materials and Methods

### 4.1. Patients

Eleven patients, who were diagnosed with advanced melanoma and treated with nivolumab between 2014 and 2017 at the Department of Dermatology, University of Occupational and Environmental Health, were enrolled in a previously published study [45]. Tumors were classified according to the 2009 American Joint Committee on Cancer (AJCC) staging system. Clinical responders and non-responders were determined based on response evaluation criteria in solid tumors (Response Evaluation Criteria in Solid Tumors, Version 1.1 (RECIST, v1.1)).

### 4.2. Flow Cytometric Analysis

PBMC samples were collected from patients with advanced melanoma as previously described [45,46]. In brief, PBMCs were isolated from the heparinized venous blood of patients by using Ficoll-Hypaque (Sigma Chemical Company, St. Louis, MO, USA) density-gradient centrifugation. After incubation for 30 min at 4 °C with monoclonal antibodies (mAbs), cells were washed twice and analyzed with a FACSCanto (BD Biosciences, San Diego, CA, USA) and FlowJo software (TreeStar, San Carlos, CA, USA). The following fluorescent-labeled monoclonal antibodies were used for surface staining: APC-conjugated anti-CD4, anti-CD11c mAbs, and anti-CD16 mAbs (BD Biosciences); APC-Cy7-conjugated anti-CD19 mAb (BD Biosciences); FITC-conjugated anti-CXC chemokine receptor 3 (CXCR3) (R&D systems, Minneapolis, MN, USA), anti-CD45RA, anti-PD-1, and anti-Lin-1 (BD biosciences, San Jose, CA, USA) mAbs; PE-conjugated anti-CC chemokine receptor 4 (CCR4), anti-CD45RO, anti-CD56, and anti-CD123 (BD biosciences) mAbs, PerCP-conjugated anti-HLA-DR mAb (BD Biosciences), and PE-Cy7-conjugated anti-CD8 mAb (BD Biosciences).

### 4.3. Microarray Data Analysis

PD-L1 expression in various organs was analyzed by a public data set deposited at the National Center for Biotechnology Information (NCBI) Gene Expression Omnibus (GEO) database (GEO accession no. GDS3834) [47], and gene expression level was examined. mRNA was extracted from human tissues, which were purchased from commercial vendors, including Clontech, Ambion, and Biochain, and subjected to microarray analysis.

To evaluate PD-1/PD-L1 and immune profiles in healthy subjects and melanoma patients, we used a total of 3 public data sets to visualize the influences of the melanoma-bearing condition on these profiles in both tumors and peripheral blood. As for the tumor side influences, we analyzed the tumor side influences on gene expression by using a public data set in 2 healthy subjects, 2 patients with nevus pigmentosus, 2 patients with melanoma in situ, 2 patients with vertical growth-phase melanoma, and 2 patients with metastatic growth-phase melanoma by using a data set (GEO accession no. GDS1989) [48]. Furthermore, we also analyzed the difference in gene expression in the tumor between primary and metastatic melanoma. Thirty-one patients with primary melanoma and 52 patients with metastatic melanoma were analyzed (GDS3966) [49]. As for systemic influences, we used a public data set to analyze peripheral blood immune profiles in 6 healthy subjects and 6 patients with melanoma (GDS2735) [50].

### 4.4. Statistical Analysis

All statistical analyses were carried out using GraphPad Prism 6.05 (GraphPad Prism Software Inc., La Jolla, CA, USA). Student’s *t*-test was used to calculate significant differences between the two groups. All *p*-values less than 0.05 were considered statistically significant.

## Data Availability

Not applicable.
